# An Effective Self-Powered Piezoelectric Sensor for Monitoring Basketball Skills

**DOI:** 10.3390/s21155144

**Published:** 2021-07-29

**Authors:** Chongle Zhao, Changjun Jia, Yongsheng Zhu, Tianming Zhao

**Affiliations:** 1 Physical Education Department, Northeastern University, Shenyang 110819, China; 2071367@stu.neu.edu.cn (C.J.); 2001276@stu.neu.edu.cn (Y.Z.); 2 College of Sciences, Northeastern University, Shenyang 110819, China; zhaotm@stumail.neu.edu.cn

**Keywords:** self-powered, PVDF thin film, motion monitoring, sport big data

## Abstract

Self-powered piezoelectric sensor can achieve real-time and harmless monitoring of motion processes without external power supply, which can be attached on body skin or joints to detect human motion and powered by mechanical energy. Here, a sensor for monitoring emergent motion is developed using the PVDF as active material and piezoelectric output as sensing signal. The multi-point control function enables the sensor to monitor the sequence of force order, angle change, and motion frequency of the “elbow lift, arm extension, and wrist compression” during shooting basketball. In addition, the sensor shows can simultaneously charge the capacitor to provide more power for intelligence, typically Bluetooth transmission. The sensor shows good performance in other field, such as rehabilitation monitoring and speech input systems. Therefore, the emerging application of flexible sensors have huge long-term prospects in sport big data collection and Internet of Things (IoT).

## 1. Introduction

Basketball gains global popularity ever since its invention in the 1890s. The shooting score determines the outcome of basketball game. Shooting accuracy is influenced by athletic experience, shooting distance, fatigue, and defensive interference. More importantly, goal percentage is closely associated with the coordination of player forearm, wrist, and fingers [1,2,3,4,5]. The shooting shots mainly consist of three technical components: elbow lift, arm extension, and wrist compression. However, in previous monitoring shooting techniques there are effect limitations on the monitoring and shooting skill analysis [6,7,8,9,10,11,12,13]. For example, previous studies have monitored changes in joint angles of shoulder, elbow, wrist, and during basketball shooting and the related multiple freedom degrees. Usually, the high-speed infrared point-of-light motions and the three-dimensional force plate are utilized to capture body motion and measure kinematic parameters, respectively. The two-dimensional fixed-point videography has also been taken to measure kinematic parameters during shooting. However, the two-dimensional videography can only acquire the angular changes in each joint in the sagittal plane, it is impossible to monitor the angular changes in large joints such as elbow and in small joints such as fingers. Multi-dimensional observation requires multi-point monitoring, which is dependent on the use of multiple devices that are usually large and expensive [14,15,16,17].

In most sensors for monitoring basketball skills, direct-current (DC) power supplies are required which is not portable. In recent years, rechargeable batteries have been developed to accelerate motion monitoring development to an extent, but they are still faced big challenges [18,19,20]. For instance, the recharging may cause device wastage and generate electronic waste. Therefore, it has been desired to develop a portable and real-time motion monitoring device without external power supply. With the development of IoT revolution, numerous flexible electronic devices have been displayed. Especial, the devices can be attached on human skin to establish human-computer interaction and achieve real-time transmission of big data [21,22,23,24,25,26,27]. It is worth noting that these multi-purpose sensors can be applied in both daily life and the field of motion monitoring [28,29,30,31,32,33,34,35]. Due to the flexibility, these devices can be portably and harmless to be attached on human skin. More importantly, these devices can realize real-time transmission and analysis of motion without external battery, and the device size can be easily designed according to actual demand [36,37,38]. Due to mechanical flexibility, lightweight, high piezoelectric output and environmental protection, PVDF is always regarded as a candidate for Nanogenerators [39,40,41].

In this study, based on the piezoelectric effect of PVDF, a flexible and wearable self-powered sensor has been presented [42,43,44,45,46]. This device can not only collect human body mechanical energy, but also can be regarded as a sensor for monitor motion state. Furthermore, the body mechanical energy can be transferred into electric energy to charge the capacitor, supplying power source for smart equipment, such as Bluetooth. Meanwhile, the piezoelectric output is unrelated with temperature. And the output piezoelectric voltage is stable at the temperature range from 26.1 °C to 46.3 °C. After the damage, it can still work by simple repairing, which exhibits the potential applications in high strength confrontation sport monitoring. Finally, we prove that the flexible and self-powered sensor can be used to monitor shoot movement in the basketball game, rehabilitation monitoring and speech input system. Above all, the flexible self-powered sensor has enormous potential in sport big data collection and Internet of Things (IoT).

## 2. Experimental Section

### 2.1. Fabrication of Sensors

Here, the sensors can be tailored according to the actual application. 15% and 85% by mass fraction of PVDF and dimethylformamide solution are stirred in water bath at 60 °C for 2 h at least. PVDF and dimethylformamide solution is set in a vacuum drying oven to remove bubble at room temperature (0.09 MPa for 40 min). Secondly, PVDF and dimethylformamide solution is dropped on the clean bottom silicon rotating surface. The spinning speed is set at 3000 r/min for 60 s. Then, PVDF/Dimethylformamide mix is dried at 90 °C for 10 min. After spinning process repeats three times, PVDF film is dried at 120 °C for 12 h, whose thickness is ~60 μm. Then 300 nm Ag layer is deposited by e-beam evaporator on both surfaces of the PVDF. After that, PVDF film is deal with polarization at 90 °C and 20 kV/mm with oil bath. Finally, PET (~100 μm) is used to be the protective layer to package the polarized PVDF film. The two wires are connected with top and bottom electrodes, respectively. Then top and bottom PET protective layers are pasted tightly to package and protect PVDF film by a sealing and cutting machine (QL5545-1 BS-D4525 Tunnel).

### 2.2. Characterization and Measurement

The morphology and structure of sensor is performed from optical microscopy (Sunny Instruments Co., LTD, SDPTOP-CX40M, Ningbo, China). The sensor performance with body motion or improved stepping motor are tested by oscilloscope (sto1102c, Shenzhen, China).

## 3. Results and Discussion

**Figure 1:**
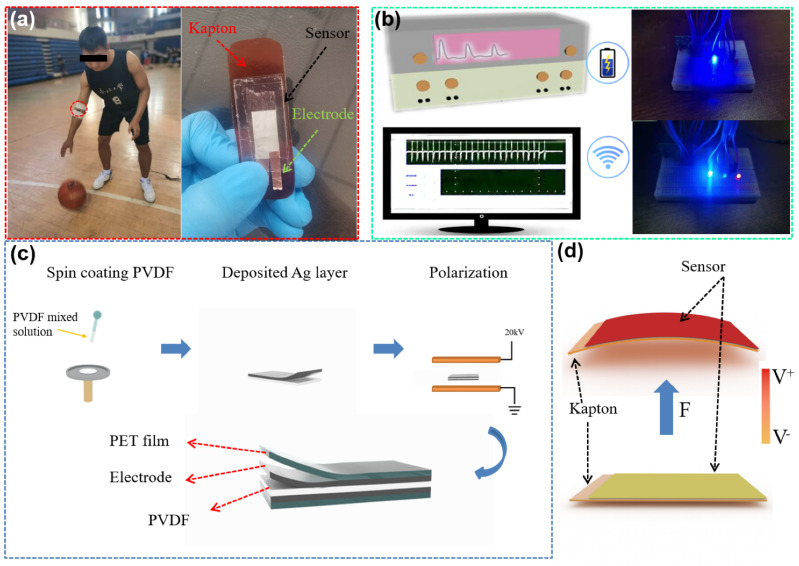
(**a**) Actual application scenario and optic image of the sensor with corresponding basic structure. (**b**) Piezoelectric signal acquisition and wireless Bluetooth transmission. (**c**) Sensor production process. (**d**) Working process of piezoelectric sensor.

The actual application scenario with optic image of the sensor, working process and mechanism are shown in Figure 1. The sensor can be attached on the joint, such as the inside elbow in Figure 1a. The optical image in the right of Figure 1 shows the structure of sensor. Sensor can monitor the angle and frequency changes in real time without power supply (driven by motion energy), and output the piezoelectric voltage (collected by oscilloscope). Based on the piezoelectric effect, these signals are not only sensing signals, but also can be used as energy to power capacitor equipment. As shown in Figure 1b, it is used to transmit sport big data for micro electronic devices. Figure 1c shows the production process of sensor. PVDF films are made by spin coating. The thickness of PVDF film is related to the number of spin coating (the experimental part for details). Figure 1d shows the working process of sensor, and the working mechanism is piezoelectric effect. When the deformation is not applied on the sensor, the dipoles in PVDF are orderly arranged, and a large number of charges are bounded on the surfaces due to the build-in electric field. When the deformation occurs, the dipole direction is changed, and the built-in electric field is reduced, releasing the surface bound charges. The output signal can be detected in the external circuit [34,35,36,37,38]. Finally, when the deformation disappears, the dipoles return to the original state and the released charges are rebounded to the surface again. The opposite signal can be detected in the external circuit.

**Figure 2:**
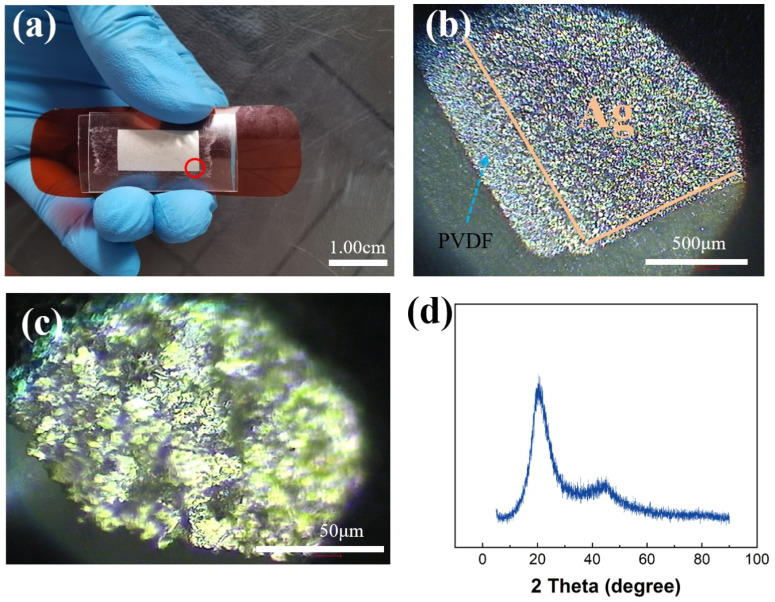
(**a**) Optical image of the sensor. (**b**,**c**) The topography of the sensor under different magnifications under an optical microscope. (**d**) XRD graph for PVDF.

The structure of sensor is shown in Figure 2a. Because PVDF film is easily damaged, we use PET to encapsulate it. To ensure that the sensor can work normally under various conditions. And stick the sensor on the kapton board to adapt to the changes in the angle of each joint of the human body. Figure 2b,c shows the appearance of the sensor is observed on microscopes with different magnifications. The silver layer on both sides of the transparent PVDF can be clearly seen. Figure 2d shows the XRD image of PVDF. There is a sharp peak at 20.6°, and there is a steamed bread peak neat 45° which corresponds β phase PVDF [47]. When the temperature is 25 °C, the resistivity of PVDF is 2.7 × 10^−1^ Ω/cm and the permittivity of PVDF is 2.9.

**Figure 3:**
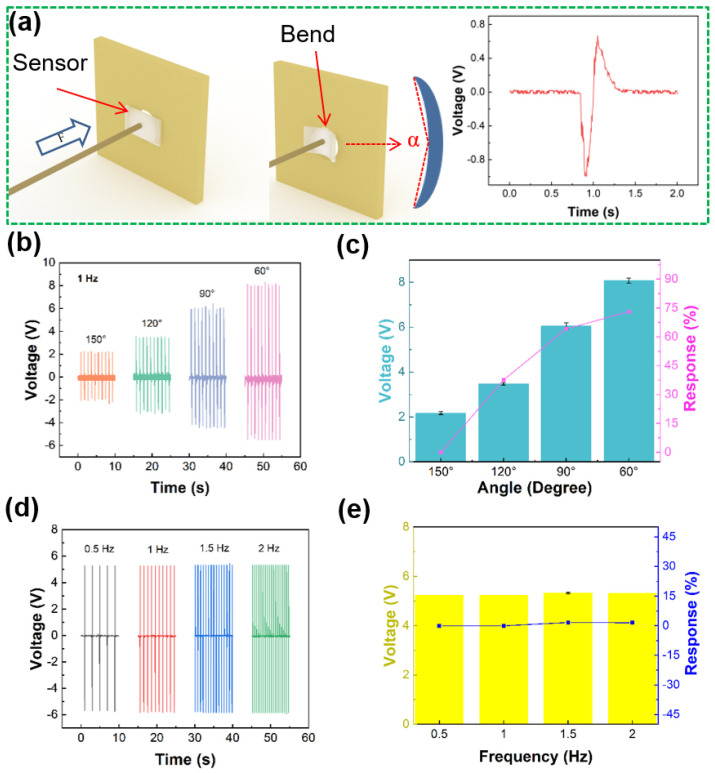
Sensing ability test of sensor. (**a**) Schematic diagram of sensor deformation and output voltage. (**b**) Output piezoelectric voltage at different working angles of sensor. (**c**) Output piezoelectric response. (**d**) Output piezoelectric voltage at different operating frequencies. (**e**) Piezoelectric output response.

The excellent sensing ability of sensor is critical factor for the practical application, as showed in Figure 3. In the sport training, the motion of joint can be similar regarded as one-dimensional motion. Therefore, a standard mechanical system in one dimension is used for simulating joint movement. Figure 3a shows the deformation process of sensor and piezoelectric voltage generated by single deformation. The sensor is fixed on the stepper motor to test under different bending angles with the same frequency or different frequencies under the same bending angle. The results show that the piezoelectric voltage generated by sensor can not only be used as sensing information, but also can charge the capacitor. Figure 3b shows the output piezoelectric voltage of sensor under different bending angles with same deformation frequency. The output piezoelectric voltage values of sensor are 2.172, 3.48, 6.052 and 8.08 V, when the angles are 150°, 120°, 90° and 60°, respectively. It shows that the output piezoelectric voltage increases with decreasing bending angle of sensor. Figure 3c shows the output piezoelectric voltage response by sensor at different bending angles, and the response of sensor can be calculated from the following Equation:

(1) $$R\%=\left|\frac{V_{0}-V_{i}}{V_{i}}\right|\times 100\%,$$

where *V*_0_ and *V_i_* are the output and piezoelectric voltages, respectively. When the angles are 176°, 174°,172° and 170°, the piezoelectric voltage responses of sensor are 0, 25, 36.3 and 43%, respectively. The results show that the biosensor can effectively sense the deformation angle. Figure 3d shows the output piezoelectric voltage of sensor at different frequencies under the same bending angle. When the frequency values are 0.5, 1, 1.5 and 2 Hz, the output voltages are 5.24, 5.24, 5.328 and 5.32 V, respectively. Moreover, the output piezoelectric voltage is relatively stable. It can be inferred that the peak value of the output voltage may be affected by the deformation angle, and the output frequency of the piezoelectric signal depends on the deformation frequency. Figure 3e shows the output piezoelectric voltage response of sensor at the same deformation angle with different frequencies. When the frequencies are 0.5, 1, 1.5 and 2 Hz, the responses of the piezoelectric voltage are 0, 0, 1.7 and 1.5%, respectively. Therefore, the sensor can monitor the change of motion angle and motion frequency. For example, it can monitor the coordination of forearm, wrist and fingers in basketball shooting or monitor the joint angle changes of important technical links.

**Figure 4:**
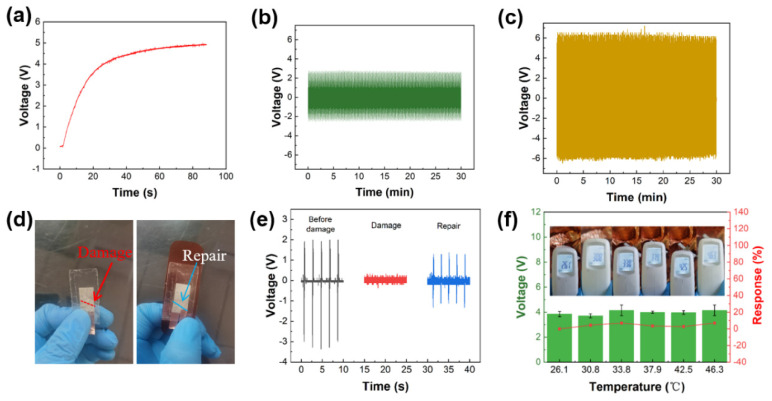
Performance test of the sensor. (**a**) Capacitor charging. (**b**,**c**) Durability test at different conditions. (**d**) Complete and damaged images of the sensor. (**e**) Comparison of output piezoelectric voltage of the damaged sensor. (**f**) Temperature test.

Figure 4 shows performance of sensor in sport scenes. Figure 4a shows that the sensor charges the capacitor. Under the condition of 1.25 Hz bending frequency with 176° angle, the 4.7 μf capacitor can be charged to 4.83 V in 90 s. Figure 4b,c shows the sensor durability and stability at different conditions. We consider that the competition time of an NBA basketball game is at least 48 min. We test the durability of sensor for 30 min. That is to say, the sensor works continuously for 30 min at a bending frequency of 1.25 Hz and an angle of 135° (as shown in Figure 4b) and a bending frequency of 2 Hz and an angle of 90° (as shown in Figure 4c), and the output piezoelectric voltage is stable. Basketball is an antagonistic sport. During the movement, the fierce confrontation may cause sensor damage. So we use scissors to cut 0.7 cm at the edge of the device in 45° to simulate the damaged state of the device during the collision (Figure 4d). In the repair process, we used epoxy resin adhesive to adhere the damaged part simply. Figure 4d shows the images of the sensor before and after repairing. As shown in Figure 4e, When the sensor is bent for five times (178°), the output piezoelectric voltage of the sensor before damaging is 1.92, 2, 1.92, 2 and 2 V, respectively. The output piezoelectric voltage of the sensor after damaging is ~0. The output piezoelectric voltage of the sensor after repairing is 1.28, 1.28, 1.36, 1.28 and 1.28 V, respectively. And the piezoelectric voltage can be up to ~65% comparing to the original piezoelectric voltage. It shows the reparability of our device under applicable conditions. Environment temperature can influence muscle sticky resistance, which can influence player’s sport performance. Human body’s temperature usually maintains 36.1–37.8 °C at heat generation and dissipation. The high temperature environment may hurt body and brain seriously when human exercises. And when heat exhaustion or heatstroke happens on player in this situation, it may endanger athletes’ lives. Therefore, we test output piezoelectric voltages of sensor when temperature is from room temperature to 46.3 °C as shown in Figure 4f. When the temperature is 26.1 °C, 30.8 °C, 33.8 °C, 37.9 °C, 42.5 °C and 46.3 °C, the output piezoelectric voltage values of sensor are 3.859, 3.702, 4.145, 3.995, 3.965 and 4.145 V, respectively. The piezoelectric voltage responses of sensor are 0, 4.3%, 6.9%, 3.4%, 2.7% and 6.9%, respectively. This results indicate that temperature has no influence on output of this sensor.

**Figure 5:**
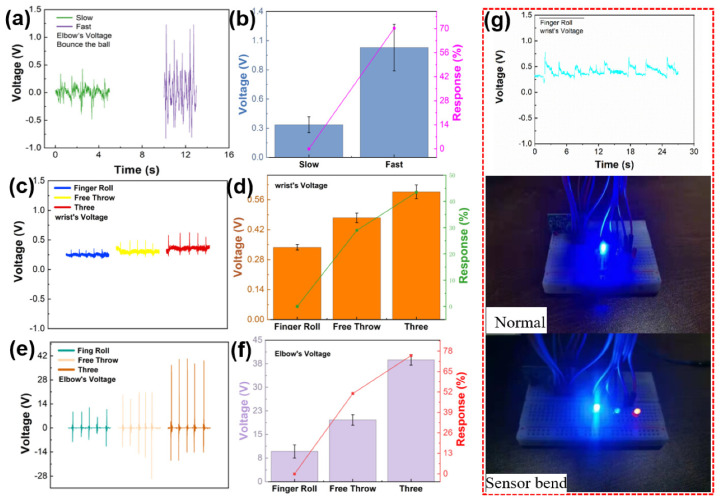
Practical application of the sensor. (**a**) Output piezoelectric voltage of ball bouncing. (**b**) Output piezoelectric voltage response of wrist joint during bouncing. (**c**) Output piezoelectric voltage of wrist joint during shooting. (**d**) Output piezoelectric voltage response of wrist joint during shooting. (**e**) Output piezoelectric voltage response of elbow joint during shooting. (**f**) Output piezoelectric voltage response of elbow joint during shooting. (**g**) Drive simple Bluetooth equipment.

The coordination of forearms, elbow, wrist and fingers is the key factor of high shooting percentage. It is particularly important to monitor and analyze the technical motions of these parts during shooting. Figure 5 shows the real-time monitoring of elbow and wrist joints by sensors during shooting. Figure 5a shows the elbow output piezoelectric voltage monitored by sensor during the slow and fast bounce motions of tester (5 times). We found that five slow bounces took longer time than those of five fast bounces. The technique of fast bouncing is fast and powerful, and the elbow angle changes greatly. Figure 5b shows the piezoelectric voltage response of slow and fast bounce. Where *V*_0_ and *V_i_* are the output of slow and fast bounce respectively, the output piezoelectric voltage responses of slow bounce and fast bounce are 0% and 70.2%, respectively. Figure 5c shows the wrist output piezoelectric voltage of the tester under the basket at two-point free throw line and three-point shooting position (5 times). The piezoelectric voltages are 0.338, 0.475 and 0.597 V. Figure 5d shows the wrist output piezoelectric voltage response of the tester is under the positions of basket, two-point free throw line and three-point line shooting. Where *V*_0_ and *V_i_* are the output of basket and the output piezoelectric voltage response of other frequencies are 0, 28.9 and 43.4%. The above data shows that the wrist angle increases with the distance of shooting, and the motion speed does not change. Figure 5e shows the output piezoelectric voltage of the elbow joint when the tester shoots under the positions of basket, two-point free throw line and three-point line shooting (5 times). The piezoelectric voltages are 9.6, 19.6 and 38.7 V, respectively. Figure 5f is the response of the elbow output piezoelectric voltage of the tester at the shooting positions of the basket, two-point free throw line and three-point line. Where *V*_0_ and *V_i_* are the output of basket and the output piezoelectric voltage response of other frequencies are 0, 51 and 75.2%. Compared with the two-point free throw line and the basket shooting position, the elbow joint output piezoelectric voltage of the three-point shooting is the largest. The reason may be that shooting from a longer distance increases the angle between the big arm and the small arm. The above data shows that when tester shooting, the motion speed (frequency) of each joint is basically the same, and the angle of elbow joint and wrist joint is related to the shooting distance. Compared with other works [48,49], we find that the sensor has the self-powered, soft, high response advantages, and we do damage simulation tests. The application value of the sensor is verified. Figure 5g shows a wireless Bluetooth transmission device driven by the sensor. The sensor monitors wrist joint output piezoelectric voltage of hook shooting under basket (10 times) to supply power for wireless Bluetooth device. It can be seen from Figure 5g that when the wrist joint does not change, the Bluetooth device does not receive the transmission signal, and the LED bulbs are in standby state. In the process of shooting, the bending sensor generates piezoelectric signals, and the LED bulbs of the wireless Bluetooth device lights up at this time (Movie S1 in Supplementary Materials). This experiment demonstrates that sensor can supply power to electronic devices and transmit data while monitoring motion, which provides more possibilities for sport big data transmission and application in the field of Internet of Things. In Movie S2, the device is attached to the wrist joint of the athlete, and the sensor is connected with the oscilloscope through wires for collecting data. When the player dribbles the ball in situ, the joint drives the sensor to produce piezoelectric output. When the motion stop, the piezoelectric output also stops. Therefore, the technical changes of athletes can be judged according to the piezoelectric voltage output. In the sport training, although the human body does multidimensional movements in frontal plane, sagittal plane and horizontal plane, multidimensional movements are made up of many one-dimensional motions of human joints. For example, shoulder joint, elbow joint and wrist joint are moving in the sagittal plane around the frontal axis. Therefore, we only tested one-dimensional motion of joints and more complex movements process are our next monitoring direction.

**Figure 6:**
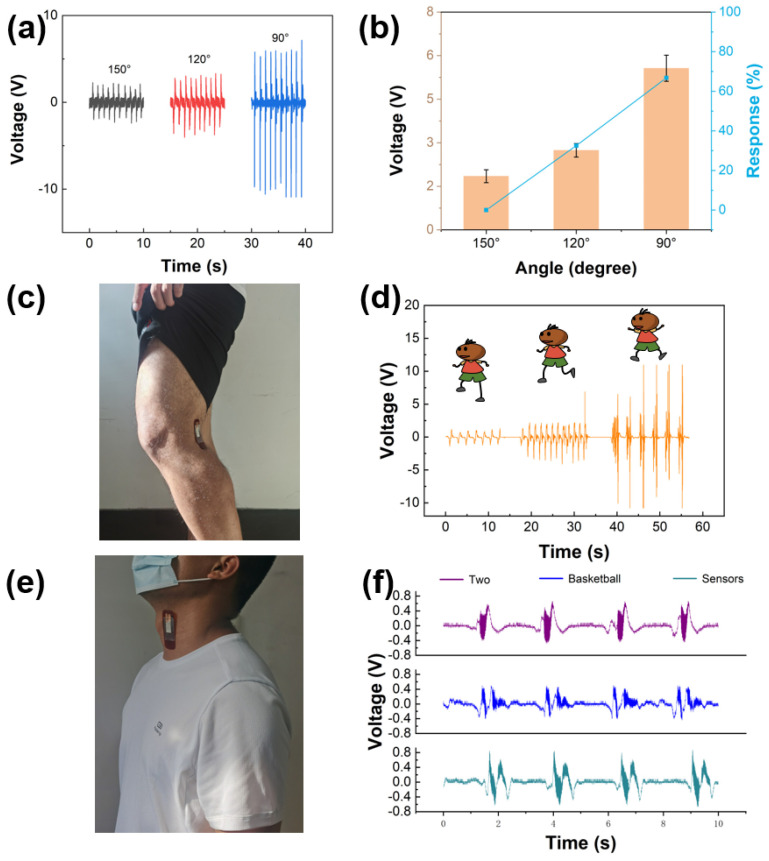
(**a**) Output piezoelectric voltage of accuracy experiment. (**b**) Output piezoelectric voltage response of accuracy experiment. (**c**) Optical image of the sensor is attached on the popliteal fossa. (**d**) Output piezoelectric voltage of popliteal fossa of sensor as the tester is walking, running, and jumping. (**e**) Optical image of the sensor is attached on the adam’s apple. (**f**) The different signals output by the sensor when tester says different words.

We use a standard mechanical system to test the performance of the device, whose bending angles and working frequencies can be controlled by program. And in the practical test, sensor is attached to joint tightly, connecting with oscilloscope through the conducting wires of sensor. Comparing Figure 6a,b with Figure 3b,c, the results in the laboratory have the same trend as those in the practical testing process. As the bending angle decreases, the piezoelectric voltage increases. The results show that the sensor which we design has a big broad application prospect in the field of sports. The potential application of the sensor in other physical motion monitoring is also demonstrated. As shown in Figure 6c, piezoelectric sensor is attached to the popliteal fossa to monitor the motion state of the knee joint. Knee joint is one of the most important power chains in human movement system, and many movements involve the movement of knee joint. When the human body is in different events or different stages of the same event, body needs to do movements such as walking, running and jumping. Figure 6d shows the output piezoelectric voltage of sensor which is attached to tester’s popliteal fossa when the tester is walking, running and jumping. The piezoelectric voltages are 1.02, 2.04 and 9.387 V, respectively. In normal walking and running movements, if the knee is not bent intentionally, the bending angle of the knee is relatively small, therefore, the output voltage of the sensor is small also. Hence, the signal is in line with the actual movement state of the human body. The knee joint is also the largest and most complex joint in the human body and is one of the most common damaged joints. The sensor also has application value in the field of rehabilitation, such as monitoring the range of motion that the joint angle can reach during the rehabilitation of the knee joint. In order to demonstrate the feasibility of the sensor as a speech input system. Sensor is installed on the throat of tester to collect voice signals (Figure 6e). As shown in Figure 6f, when the tester says three different words: “two”, “basketball”, and “sensors”, the sensor outputs three different voltage signals. The sensor is able to recognize small changes in pronunciation and shows good performance. The above results also prove the great potential of the sensor in motion monitoring, human-computer interaction and other fields.

## 4. Conclusions

To sum up, a kind of self-powered sensor based on the PVDF piezoelectric effect is proposed. The sensor can be fully attached to the surface of the human body. As the sensing device, it can monitor the movement frequency and movement angle of players in different movement states. At the same time, it can also be used as a sensing input device in rehabilitation monitoring and voice input system, displaying various sensing functions. Meanwhile, it can convert motion mechanical energy into electric energy, so it can charge micro electric equipment such as Bluetooth device. The sensor has the characteristics of anti-damage and easy repair. In the use process, it is less affected by the factors of temperature and use times. This work not only provides a promising method for sports big data collection but also promotes the application and development of flexible self-powered sensors based on piezoelectric nanogenerators.

## Data Availability

The data presented in this study are available on request from the corresponding author.

## Supplementary Materials

The following are available online at https://www.mdpi.com/article/10.3390/s21155144/s1, Movie S1: The self-powered piezoelectric sensor can transmit wireless signal to control the LED, Movie S2: The practical test movie, Table S1: The self-powered Portable Flexible Sensor comparison with previous works.
